# Plant species richness response to atmospheric nitrogen deposition across bedrock types in the United States and Czechia

**DOI:** 10.1002/ecs2.70663

**Published:** 2026-05

**Authors:** Tomáš Chuman, Christopher M. Clark

**Affiliations:** 1 Department of Physical Geography and Geoecology, Charles University, Prague, Czech Republic; 2 Czech Geological Survey, Prague, Czech Republic; 3 Integrated Environmental Assessment Branch, US Environmental Protection Agency, Washington, DC, USA

**Keywords:** atmospheric nitrogen deposition, bedrock, critical loads, Czech Republic, non-native species, plant species richness, United States

## Abstract

Atmospheric nitrogen (N) deposition leads to many changes in terrestrial and aquatic ecosystems, affecting ecosystem processes and species composition. Terrestrial vegetation often shifts from being N-limited to light-limited and becomes dominated by a few fast-growing strong competitors and generalist species. However, once vegetation is not N-limited, other nutrients such as phosphorus or base cations might limit growth, constraining the expansion of strong competitors. Using extensive data from the United States (US) and Czech Republic (CZ) for open-canopy (herbaceous vegetation and shrublands) and closed-canopy (forest) vegetation, we examined differences in response to N deposition arising from either base cation-rich, moderate, or poor bedrock. We hypothesized that increased N deposition on cation-poor bedrock could have a weaker effect on plant species richness changes than on cation-rich bedrock, because under the multiple element limitation paradigm, the expansion of strong competitors and competitive exclusion could be limited by other nutrients in a cation-poor environment. Our results, after controlling for other environmental factors, including S deposition, show that the effect of N deposition on species richness differs by bedrock type and plant species richness declines the most on cation-rich bedrock as hypothesized, except for open-canopy vegetation in the United States. We found a canonical unimodal relationship to N deposition for both vegetation types in both countries. Although species richness generally shows an initial increase at low nitrogen deposition loads before declining, it decreases steadily across the full nitrogen deposition gradient in both vegetation types occurring on cation-rich bedrock in the Czech dataset. Although the species richness change is driven by the interplay among several factors, the same response of species richness to N deposition and the same derived critical N deposition values, ranging from 8 to 14 kg N ha^−1^ year^−1^, across such a broad gradient in both countries, suggest the robustness of the results. Overall, the results also agree with the empirical critical loads defined for the US ecosystems and European ecosystems, but suggest that limitation by cations or other nutrients may play a role as well in the response to N deposition.

## INTRODUCTION

Though nitrogen (N) is an essential plant nutrient (Vitousek et al., 1997), N deposition, which may be high in many industrialized nations due to N emissions from fossil fuel combustion and application of synthesized agriculture fertilizers and manure (Fowler et al., 2013; Galloway, 2003; Vishwakarma et al., 2023), leads to many changes in terrestrial and aquatic ecosystems. The changes in terrestrial ecosystems include changes in (1) total N availability, (2) relative availability in reduced or oxidized forms (Moldan et al., 2006; Oulehle et al., 2018), (3) soil properties including pH, base saturation and thus nutrient availability (Aber et al., 1998; Bowman et al., 2008; Lovett et al., 2009), and (4) in microbial composition and activity (Freedman et al., 2013; Morrison et al., 2018; Oulehle et al., 2018). All of these changes affect ecosystem processes and species composition, thus potentially threatening biodiversity, human health, water quality, and contributing to climate change (Bobbink et al., 2010; Sutton et al., 2011). Understanding these effects is crucial for assessing N deposition’s impacts and taking appropriate abatement measures.

Excessive N input into N-limited terrestrial ecosystems increases plant biomass production (Braun et al., 2010; Fenn et al., 2003; Hautier et al., 2009; Palpurina et al., 2019; Spohn et al., 2025). Vegetation then may shift from being N-limited to light-limited (Hautier et al., 2009) and often to a dominance of fast-growing and strong competitors that may be generalist species with larger ranges (Bobbink et al., 2010; Clark et al., 2019; Staude et al., 2020, 2022). It is not only the absolute amount of N inputs but also the shift in the ratios of nutrients that was shown to be associated with loss of species diversity (Harpole & Tilman, 2007; Roem & Berendse, 2000; Stevens et al., 2011). Most plant species that are lost from the community are species from oligotrophic sites adapted to nutrient-poor conditions that typically persist on soils with low nutrient availability that are insufficient for the strong competitors. Because low N availability is much more common over evolutionary time scales compared with high N availability (mostly a recent anthropogenic phenomenon), we hypothesize that there are far fewer species adapted to high N availability conditions (Tilman, 1990). Thus, overall, the number of species in an area typically declines with higher N addition above some level.

Many studies focus on the effect of N deposition on species richness in various ecosystems. The studies are in agreement for grasslands and heathlands, showing declines in species richness and changes in species composition with increasing N availability (Brumme & Khanna, 2008; Carroll et al., 2003; Clark & Tilman, 2008; Duprè et al., 2010; Humbert & Dwyer, 2016; Jones et al., 2004; Maskell et al., 2010; Simkin et al., 2016; Stevens et al., 2004; Stevens, Duprè, et al., 2010; Zavaleta et al., 2003). However, a European study by Ceulemans et al. (2013) concluded that phosphorus enrichment presents a greater threat to grassland plant species richness than N enrichment, as total species number and the number of forbs were significantly higher in phosphorus-limited and phosphorus and N co-limited grasslands. These conclusions were later challenged by Soons et al. (2017), showing that N enrichment or combined N and phosphorus enrichment reduced plant species, not phosphorus enrichment itself. Muehleisen et al. (2023) further showed that other nutrients, such as potassium, also play an important role, and the loss of species was greatest due to shifts in the availability of nitrogen, phosphorus, and potassium together. Regardless, these studies indicate that multiple elements, including base cations, may affect the response to N addition.

Studies from forests show much less agreement on the effect of increased N availability on plant species richness of the herbaceous vegetation layer. In a gradient study, Simkin et al. (2016) examined the relationship of forb and graminoid species richness with N availability across the conterminous United States, demonstrating that the relationship was positive for very low N deposition loads (4–10 kg N ha^−1^ year^−1^) and negative at higher levels (>10–13 kg N ha^−1^ year^−1^). Contrastingly, other studies show no response of species richness to N addition (Gilliam & Hockenberry, 2006; Schleppi et al., 1999), probably due to chronically high historical and ambient N deposition at these sites, that is, the ecosystems are close to or already past N saturation. These studies were also relatively short-term, lasting 6 and 3 years, respectively. Even though the studies did not show a consistent effect of N deposition on the species richness in forests, most found that N deposition alters species composition and species vegetation cover (Dirnböck et al., 2014; Hurd et al., 1998; Landuyt et al., 2024) or triggers an increase in the number or vegetation cover of nitrophilous species (Buriánek et al., 2013; Ewald et al., 2013; Landuyt et al., 2024; Novotný et al., 2016; Roth et al., 2022). Other studies, however, conclude that these changes could be attributed to changes in light availability rather than as a direct effect of increasing N availability (Landuyt et al., 2024; Perring et al., 2018; Roth, Michiels, Puhlmann, Sucker, & Hauck, 2021; Roth, Michiels, Puhlmann, Sucker, Winter, & Hauck, 2021; Verheyen et al., 1999). In the Czech Republic (CZ), high-resolution historical atmospheric N deposition estimates were only recently made available (Oulehle et al., 2016). This made it possible to analyze the effect of N deposition on vegetation and to analyze how strong influence N has as one of the threatening factors. Because threatening factors vary for particular vegetation types (Chytrý et al., 2019); and, in many of them abandonment of traditional management is the most significant factor driving vegetation changes (Hédl et al., 2010; Klinkovská et al., 2023; Müllerová et al., 2015), the effect of eutrophication caused by atmospheric N deposition is often hard to separate from other factors (Klinkovská et al., 2024).

Nevertheless, studies generally agree that the response to N deposition is strong and context-dependent; and, there are many confounding factors that often do not act independently but rather form feedback loops. These confounding factors include forest type, soil properties such as pH, C/N ratio, base saturation, and soil moisture (Roth, Michiels, Puhlmann, Sucker, & Hauck, 2021; Roth, Michiels, Puhlmann, Sucker, Winter, & Hauck, 2021; Simkin et al., 2016), density of large herbivores tending to prefer certain species (Bernhardt-Römermann et al., 2015), land use history, management practices, duration of exposure to N deposition, and the total amount and form of N (oxidized or reduced) (Bobbink et al., 2010; Zhang et al., 2018). The form of N available to plants, which some species are sensitive to, depends strongly on soil pH. Ammonium (NH_4_^+^ ) is the dominant form of N in acid soils, whereas nitrate (NO_3_^−^) is the dominant form of N in neutral and alkaline soils (Gigon & Rorison, 1972). The utilization of NH_4_^+^ further reduces pH as H^+^ ions are excreted by the plant root upon uptake to maintain charge balance. Excess NO_3_^−^, if not taken up by plants or microbes, leaches out of the soil due to high mobility and may carry with it base cations. In industrial areas that experienced soil acidification, primarily due to high sulfur deposition loads during the 1970s–1980s, excessive nitrogen can exacerbate this acidification in soils that are still acidified or have started to recover from acidification. In poorly buffered soils, the leaching of NO_3_^−^ can also exacerbate soil acidification by removing cation buffers, and leading to nutrient imbalance and potential Al, Mn, and Fe toxicity (Aber et al., 1998; Bowman et al., 2008; Lovett et al., 2009; Oulehle & Hruška, 2005). Soil acidification itself was also shown to affect species composition and species richness significantly (Stevens, Thompson, et al., 2010). Moreover, most of the abovementioned factors interact with climate change (Antão et al., 2022; Sanczuk et al., 2024) or recovery from acidification (Tipping et al., 2021).

Due to a lack of consistent patterns of plant species richness response to N deposition across different vegetation types and broad environmental gradients, we analyzed the species (herbs and graminoids) richness response to N deposition using datasets from the United States and the CZ with different histories of land use and atmospheric deposition (Kopáček & Veselý, 2005; Moldan & Schnoor, 1992). In the CZ, most ecosystems are exposed to chronically high N deposition (>10 kg N ha^−1^ year^−1^), unlike ecosystems in the United States, where there are areas with high N deposition (>20 kg N ha^−1^ year^−1^) and areas with very low N deposition (<5 kg N ha^−1^ year^−1^; Simkin et al., 2016). A comparison of these datasets helps us understand how species richness changes across a much broader N deposition gradient, especially in areas with low N deposition, which are uncommon across most of Europe.

Published research generally shows that the soil plays an important role in the effect of N deposition on plant species richness and composition through pH, nutrient levels, their availabilities and ratios, and soil buffering capacity. However, these soil properties vary spatially and, in some cases, seasonally, and their values depend on sampling depth and the soil horizons examined. Moreover, data on soil properties are not readily available in a sufficient spatial resolution across broad scales. Therefore, we hypothesized that bedrock type—a more readily available characteristic—would constitute a better cofactor for assessing the variable effects of N deposition on plant species richness.

Thus, assuming that N deposition does not contribute significantly toward acidification, we hypothesized that species richness will show the most significant response to N deposition on the cation-rich bedrock, where limitation by other nutrients (base cations or phosphorus) will be less likely. N deposition in these cation-rich environments would lead to increased expansion of strong competitors, and species richness would thus decrease due to competitive exclusion.

However, weak plant competitors are often outcompeted by other plants, such as those favoring N or invasive species, without affecting the total number of species (Bobbink et al., 1998). Therefore, we also examined the native to non-native species richness ratio response. If non-native species outcompete native species, the native to non-native species ratio should decrease.

## DATA AND METHODS

Here, we examined the relationship between herbaceous plant species richness and N deposition across three different bedrock types in the United States and the CZ and the relationship between native to non-native species richness ratio and N deposition in the United States. We analyzed two broad vegetation types (open- and closed-canopy vegetation) separately and controlled for additional explanatory variables (Table 1) such as precipitation, temperature, sulfur (S) deposition, and vegetation sampling plot size.

### Overview of explanatory variables

The two broad vegetation types of closed-canopy (forests) and open-canopy (herbaceous vegetation and shrublands) vegetation were defined by the National Land Cover Database (Simkin et al., 2016) in the US dataset. We adopted these US vegetation classes for the Czech dataset as well. The forest vegetation from the Database of the Czech Flora and Vegetation (PLADIAS) (Chytrý et al., 2021) was assigned to closed-canopy vegetation and non-forest vegetation to the open-canopy vegetation in the Czech dataset.

To test for soil differences arising from bedrock, such as pH, nutrient levels, and soil buffering capacity, we used the Map of Geochemical reactivity of rocks of the CZ (MGeoReaCR) (Chuman et al., 2014). We characterized broad classes of base cation content and susceptibility to weathering distinguishing (1) high reactivity rocks, (2) medium reactivity rocks, and (3) low reactivity rocks. We assigned sites found on these categories to be cation-rich, cation-moderate, and cation-poor, respectively. The cation-rich sites are (1) base cation-rich on carbonaceous rocks (limestone; calcareous claystone, siltstone or sandstone, loess; dolomite) or pyroclastic deposits (basalt, basanite, tephrite, rhyolite, ignimbrite, andesite); (2) cation-moderate sites are on nutrient medium-rich or rich but slowly weathering rocks (granite, granodiorite, diorite, orthogneiss, migmatite, wacke, andesite, phonolite, trachyte); and (3) cation-poor sites are on cation nutrient-poor bedrock (non-carbonaceous sandstone, conglomerates, arkose, greywacke, quartzite, chert, loamy loess). These three categories, though intra-level variability exists, are a good predictor for water chemistry (Chuman et al., 2013), soil chemistry (Hubová et al., 2018), and a good predictor for discriminating the distribution of woodland communities (Vojta et al., 2023) in the CZ. Therefore, for the United States, we used the Global Lithological Map Database (GLiM) (Hartmann & Moosdorf, 2012) as an equivalent in a similar thematic resolution. The translation of the GLiM classes to cation levels as used for the Czech dataset is given in Table 2. (For maps of the two countries showing the distribution of these three bedrock cation levels, see Appendix S1: Figures S1 and S2).

### US species richness and explanatory variables

For the herbaceous (graminoid and forb) species richness and plot sizes (sample areas), we used the database compiled by Simkin et al. (2016) covering the continental United States from 1990 to 2013. The environmental variables (i.e., annual average precipitation and temperature normals) were both obtained from PRISM (1981–2010) (Table 1). Average total nitrogen deposition and average total sulfur deposition were obtained from the National Atmospheric Deposition Program (NADP) Total Deposition (TDep) committee (averaged over the grids from 2000 to 2012; version 2 of 2013). Total deposition was the sum of wet and dry deposition. Wet deposition was calculated by the NADP and dry deposition was the product of the NADP wet deposition and the ratio of dry: wet deposition as estimated by the Community Multiscale Air Quality (CMAQ) model (Byun & Schere, 2006). This estimate is the same as was used in Simkin et al. (2016) and was found to be highly correlated with newer updated estimates of N and S deposition (i.e., TDEP, Schwede & Lear, 2014). See Simkin et al. (2016) for additional details.

### Czech Republic species richness and explanatory variables

For the species richness data including vegetation sampling plot sizes (sample areas), we used vegetation plots from the National Phytosociological Database (Chytrý & Rafajová, 2003) sampled between 1990 and 2013, including forest vegetation (except for alder and willow carrs), alpine and subalpine vegetation, grassland and heathland vegetation (except for saline grasslands, vegetation of annual graminoids in saline habitats and vegetation of annual succulent halophytes) and scrub vegetation (except for riparian willow scrub and willow–poplar forests) as classified in the PLADIAS database (Chytrý et al., 2021). The selection of herbaceous species, to have the compatible species richness data with the US database, was based on the list of life forms (Dřevojan et al., 2023) available from www.FloraVeg.eu.

Mean annual temperature and mean annual precipitation for 1990–2013 for the sites were obtained from modeled annual temperature and precipitation raster maps (Czech Hydrometeorological Institute, 2024). Total average N deposition over the vegetation sampling period was calculated by multiplying wet N deposition (Oulehle et al., 2016) by the dry: wet deposition ratio derived from the EMEP grid maps for the CZ (EMEP, 2024). In contrast to total N deposition, which was calculated using the dry deposition factor from the EMEP data, total average S deposition over the vegetation sampling period was calculated using the model for total sulfur deposition, as described by Oulehle et al. (2016).

All vegetation plots (US and CZ) were georeferenced, and the environmental data were extracted from raster maps by overlaying the point and raster layers using ArcGIS (ESRI, 2021).

### Statistical analysis

We analyzed the US and Czech data separately for both vegetation types.

In both the Czech and US datasets, the quantitative explanatory variables exhibited moderate pairwise correlations (|*r*| ≥ 0.5) (Table 3). Still, variance inflation factor values indicated problematic multicollinearity only for the Czech dataset; therefore, to control for correlated environmental variables (precipitation, temperature, N, and S deposition) and separate their influence in the Czech dataset, we performed a principal components analysis (PCA) for the Czech dataset only with varimax rotation of standardized environmental variables (to a mean of 0 and a SD of 1), to yield uncorrelated, more interpretable components, and analyzed for the significance of these uncorrelated rotated components (RC). The PCA and the varimax rotation were performed using the *psych* package in R (Revelle, 2024).

We then used a polynomial least square regression with interactions, because we expected nonlinear relationships (e.g., Simkin et al., 2016; Stevens, Duprè, et al., 2010). The saturated model included: (1) bedrock, (2) sample area, (3) either the four original explanatory variables (US dataset) or rotated PCA components (Czech dataset), (4) quadratic terms for all continuous variables, and (5) all pairwise interactions. We calculated the corrected Akaike information criterion (AICc) values for models with every combination of explanatory variables assuming hierarchy using the *dredge* function in the *MuMIn* R package (Bartoń, 2013), and the best model was selected based on the lowest AICc value.

## RESULTS

**FIGURE 1:**
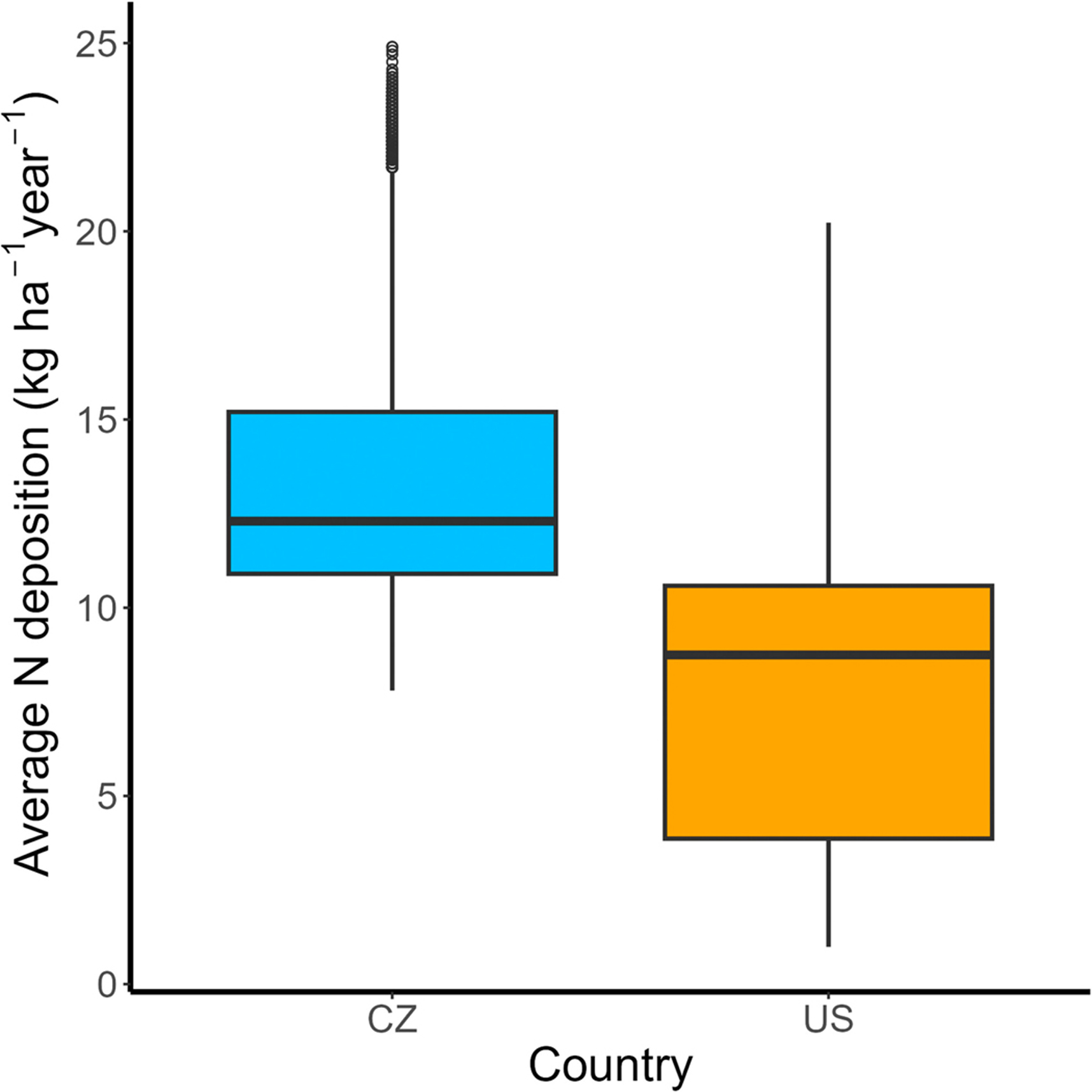
Comparison of the average N deposition for the Czech Republic (CZ) and the United States (US). The data for the CZ are average N deposition values for vegetation plots of the period 1990–2013. For the United States, these are the average N deposition values for vegetation plots of the period 2000–2012. See Table 1 for details. For the box plot comparing the same period 2000–2012, see Appendix S1: Figure S3. The box shows the interquartile range (IQR) (25th–75th percentile) with the median. Whiskers extend to values within 1.5× IQR and data points beyond are outliers.

The datasets contained 15,271 and 22,501 samples from the United States and the CZ, respectively. These data cover almost complete environmental gradients in these countries, even though geographically, the southwest is underrepresented in the US dataset (Clark et al., 2025; Simkin et al., 2016). The datasets show a different range of environmental factors, as expected (Table 4), with different N (Table 4, Figure 1; Appendix S1: Figure S3) and S deposition legacies (Table 4). Mean S deposition in the CZ was double compared to the United States, with the minimum value of approximately 4 kg S ha^−1^ year^−1^ and <1 kg S ha^−1^ year^−1^, respectively.

In the CZ, the minimum value of total N deposition was 8 kg ha^−1^ year^−1^, which is already in the range of empirical critical values for most temperate ecosystems in the CZ (Bobbink et al., 2022). In the United States, there were relatively “clean” areas with low N deposition (<2 kg N ha^−1^ year^−1^). Approximately, 75% of the US plots experienced N deposition below 75% of the Czech plots. A comparison of these datasets helps us understand how species richness changes across a much broader N deposition gradient, especially in areas with low N deposition, which are uncommon across most of Europe.

Plant species richness was generally higher for open-canopy vegetation compared to closed-canopy vegetation in the CZ, and open-canopy species richness was generally higher in the CZ than in the United States. Closed-canopy vegetation had on average the same number of species in the United States and CZ. For the United States, the average species richness of both vegetation types was similar (Table 5). Mean species richness was consistently higher for sites on cation-rich bedrock for both countries and vegetation types.

### Species richness response to N deposition in the US vegetation

**FIGURE 2:**
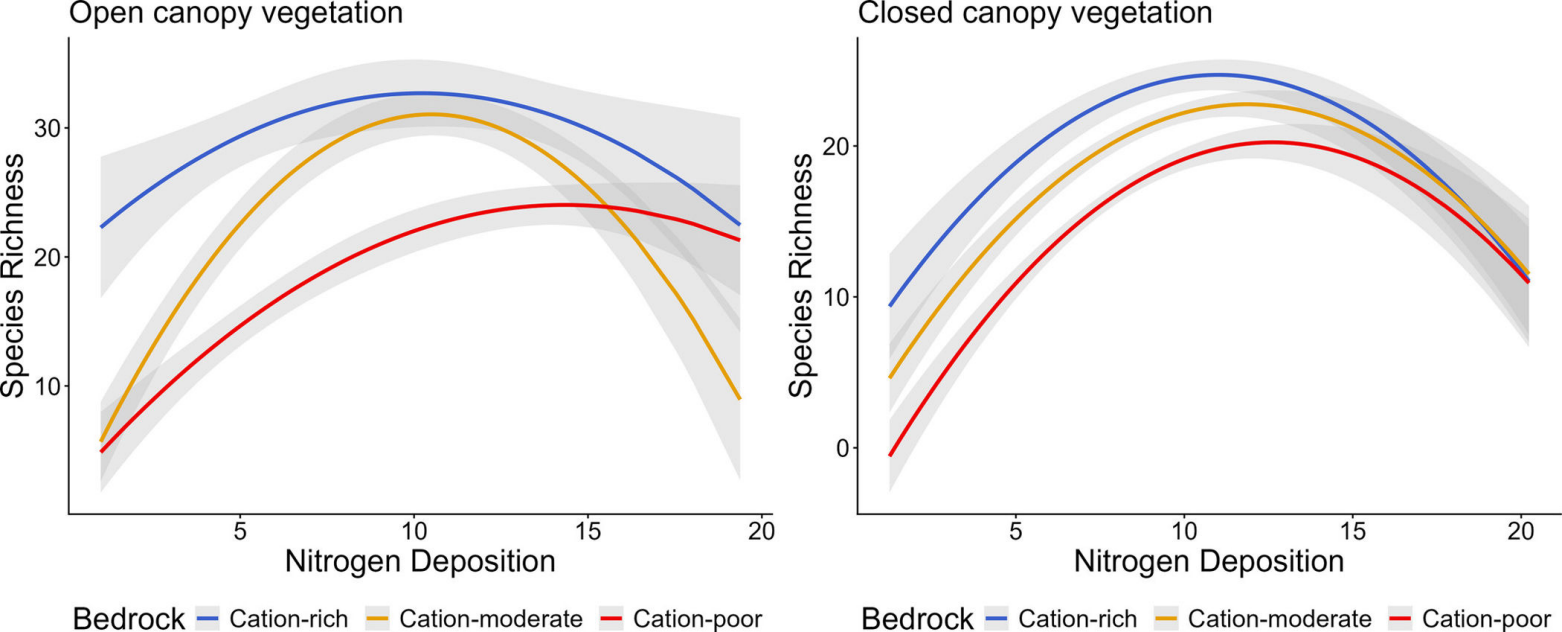
Predicted values of the species richness for open- and closed-canopy vegetation in the United States along the average N deposition gradient on different bedrocks. The shaded bands represent 95% CIs around the predicted values.

We found that species richness was significantly and unimodally related to N deposition for both vegetation types after controlling for the other environmental variables and sample area, and that response differed by bedrock. The unimodal relationship between species richness and N deposition showed both positive and negative effects of N deposition. The models explained 47% and 17% of the variance for the open- and closed-canopy vegetation, respectively, in the United States (open: adjusted *R*^2^ = 0.47, *F*_30,3475_ = 103.7, *p* < 0.001; closed: adjusted *R*^2^ = 0.17, *F*_27,11,737_ = 92.22, *p* < 0.001) (Appendix S1: Table S2). For the open-canopy vegetation, in the selected model species richness showed a significant nonlinear relationship with N deposition, with both the rate of change and the shape of the response varying across bedrock types. For the closed-canopy vegetation, the best model did not contain the interaction of the bedrock and quadratic term of the N deposition, suggesting that species richness exhibited a significant nonlinear response to N deposition, also the rate of change differed among bedrock types, but the overall curvature of the response was similar across them (Figure 2). There were six competing models for the open-canopy vegetation and three competing models for the closed-canopy vegetation with a delta AICc value <2 (Appendix S1: Table S2). The structure of variables in these candidate models was very similar, and these models always contained the N deposition and bedrock and their interaction. We found species richness increases with increasing N deposition for both vegetation types across all bedrock sites at the low N deposition loads, similar to earlier studies (Simkin et al., 2016). For the open-canopy vegetation, species richness increases up to about 10 kg N ha^−1^ year^−1^ of N deposition load for cation-rich and cation-moderate bedrock types, before the species richness declines (Figure 2) but for the cation-poor bedrock, it increases up to 14 kg N ha^−1^ year^−1^ of N deposition load. The magnitude of species changes is the greatest on cation-moderate bedrock. For closed-canopy vegetation, the species richness increases reach their peaks at a range 10–13 kg N ha^−1^ year^−1^ across all bedrock types, with the peak occurring at lower deposition loads on cation-rich bedrock and at higher loads on cation-poor bedrock, and then the species richness decreases at a similar rate (Figure 2). This suggests slightly higher sensitivity of species richness to N deposition on cation-rich bedrock compared to the other two types. Although the sites at cation-rich bedrock are generally more species-rich, the open-canopy sites at cation-moderate bedrock show the highest increases in species richness as well as the strongest declines in response to nitrogen deposition.

The pattern of species richness changes along the N deposition gradient depicted in Figure 2 should be interpreted with caution at sites where the N deposition loads exceed 15 kg N ha^−1^ year^−1^. The prediction curves have broader CIs, as there are relatively small numbers of sites that experience these high N deposition levels in the United States.

### Species richness response to N deposition in Czech vegetation

The PCA analysis helped us extract uncorrelated components and indicated (Appendix S1: Table S3) that the first two principal components explained the vast majority of the total variance (96% for both canopy types); therefore, only these two components were included in the subsequent models. However, we could not separate N and S deposition, even after varimax rotation, as N and S deposition showed high loadings on the same component. For the open-canopy vegetation, the first RC represented N and S deposition gradients; the second RC represented mean annual temperature gradient. For the closed-canopy vegetation, the first RC represented mean annual precipitation gradient; the second represented N and S deposition gradient. We found that species richness was significantly related to the RC representing N and S depositions for both vegetation types after controlling for the other environmental variables and sample area, and that response differed by bedrock. The models explained 12% and 7% of the variance for the open- and closed-canopy vegetation, respectively (open: adjusted *R*^2^ = 0.12, *F*_17,12,914_ = 104, *p* < 0.001; closed: adjusted *R*^2^ = 0.07, *F*_16,9552_ = 45.75, *p* < 0.001) (Appendix S1: Table S4). For the open-canopy vegetation, the best model contained all variables, their quadratic terms, and pairwise interactions. For the closed-canopy vegetation, the best model included all variables, their quadratic terms, and pairwise interactions, except for the quadratic term of sample area. Other than the best models with the lowest AICc, there was no competing model for the open-canopy vegetation and two competing models for the closed-canopy vegetation within the delta AICc value <2 (Appendix S1: Table S4). The structure of variables among models was very similar, and models always contained the RC representing N and S deposition gradients and its interaction with bedrock.

**FIGURE 3:**
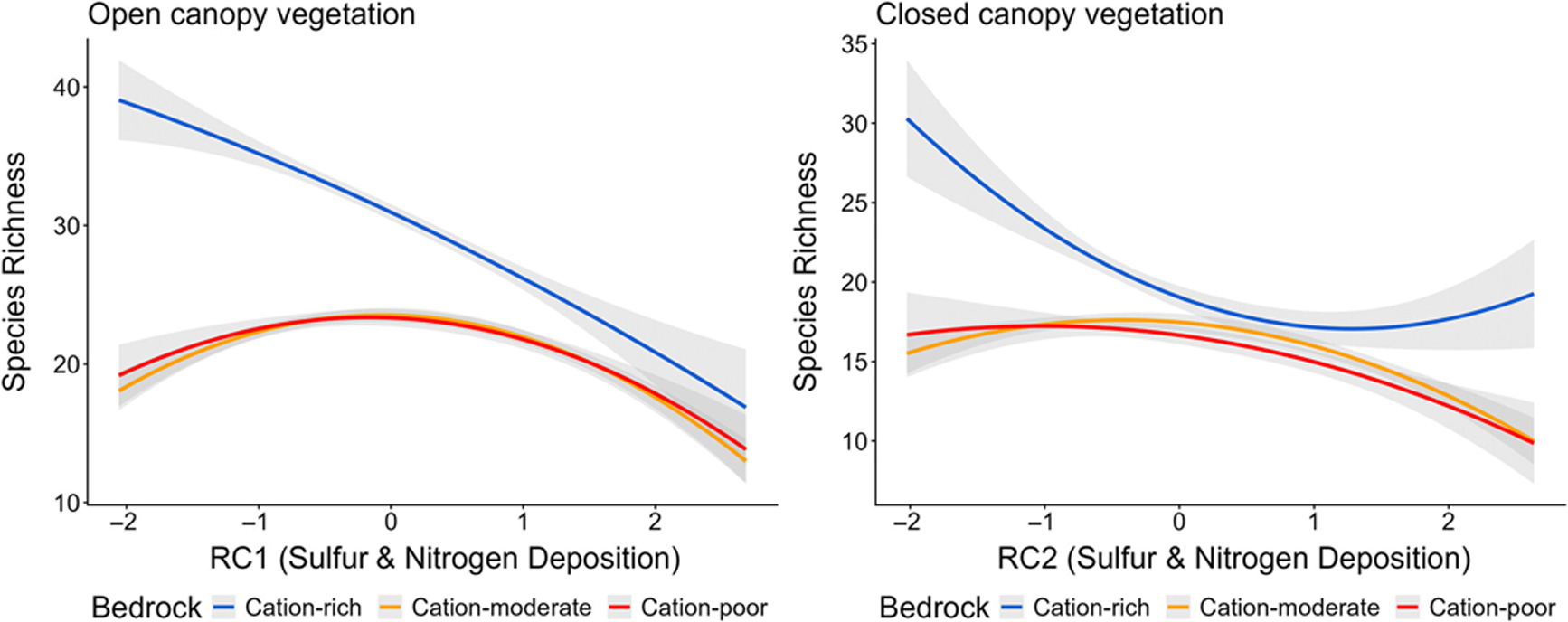
Predicted values of the species richness along the gradient represented by the first (RC1) and second rotated components (RC2) as interpreted here as the S and N deposition for open- and closed-canopy vegetation, respectively, on different bedrocks in Czechia. The shaded bands represent 95% CIs around the predicted values.

Based on the close linear relationship between RC1 (open) and RC2 (closed) and both N and S depositions (Appendix S1: Figures S4 and S5), we derived estimates of the associated N and S deposition levels for both vegetation types. For the open-canopy vegetation, we see a similar unimodal pattern in the species richness response to N and S deposition gradients (Figure 3) as for the N deposition gradient in the US dataset. In the Czech dataset, species richness increases at low deposition loads and then decreases above a threshold of 10–11 kg N ha^−1^ year^−1^ (6–7.5 kg S ha^−1^ year^−1^) for cation-moderate and cation-poor bedrock. However, species richness was higher and declined across the whole deposition gradient for cation-rich sites (Figure 3; Appendix S1: Figure S4). The cation-moderate and cation-poor bedrock show similar responses regarding the magnitudes of changes in species richness. The cation-moderate and cation-poor bedrock sites tend to have similar species richness across most of the RC2 (N and S deposition) gradient. The closed-canopy vegetation sites at the cation-moderate and cation-poor bedrock again were similar, and tended to have the lowest changes in species richness compared to cation-rich bedrock; however, the shape of the curves is inverted for the cation-rich bedrock. On cation-moderate and cation-poor sites, species richness tends to slightly increase at low deposition levels, but begins to decline at 11–13 kg N ha^−1^ year^−1^ (15–20 kg S ha^−1^ year^−1^) with an earlier onset of decline on cation-poor bedrock (Figure 3; Appendix S1: Figure S5). Species richness on cation-rich bedrock decreases from the lowest values of N and S depositions and tends to level off at 18 kg N ha^−1^ year^−1^ (40 kg S ha^−1^ year^−1^).

### Native to non-native species richness ratio response to N deposition in the US vegetation

**FIGURE 4:**
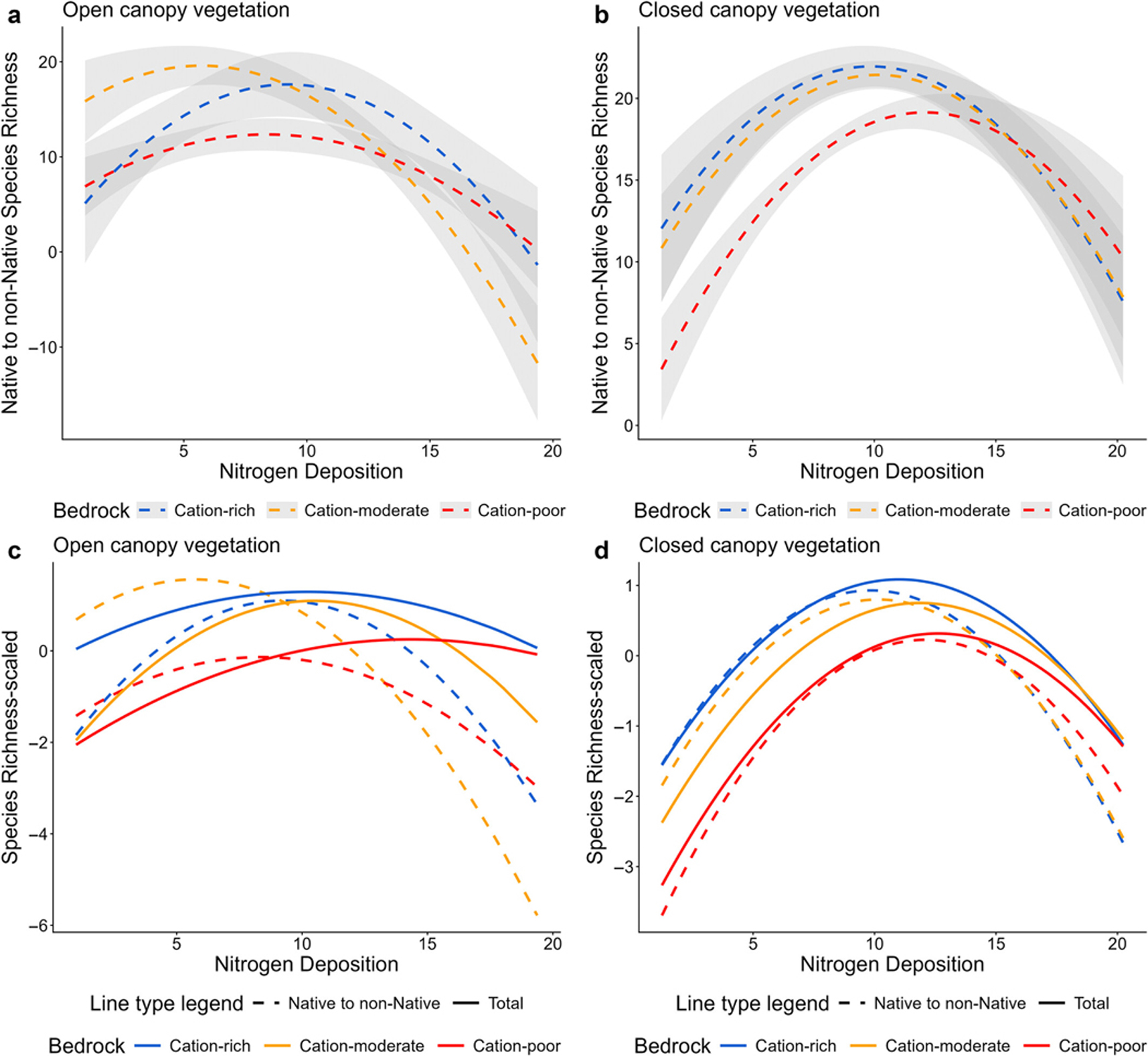
Predicted values of the native to non-native species richness ratio in the United States along the average N deposition gradient for (a) open- and (b) closed-canopy vegetation and the overlay of the predicted values for native to non-native species richness ratio and species richness for (c) open- and (d) closed-canopy vegetation. The non-native species included introduced species and those classified as invasive. The native to non-native species richness ratio and species richness in the overlaying graphs were scaled to zero mean and unit variance (*z* standardization). The shaded bands represent 95% CIs around the predicted values.

To analyze the native to non-native species richness ratio in the open and closed vegetation in the US dataset, we used the same variables as described in Data and methods: Overview of explanatory variables. We found that species richness ratio was significantly unimodally related to N deposition for both vegetation types after controlling for the other environmental variables and sample area, and the response differed by bedrock (Figure 4a,b). However, for the closed-canopy vegetation, the best model did not contain the interaction of the bedrock and quadratic term of N deposition, suggesting, similar to the US closed-canopy species richness, that the native to non-native species richness ratio exhibited a significant nonlinear response to N deposition. In addition, the rate of change differed among bedrock types, but the overall curvature of the response was the same across them (Figure 4a). The unimodal relationship between native to non-native species richness and N deposition also showed both positive and negative effects of N deposition. The best models explained 42% and 13% of the variance for the open- and closed-canopy vegetation, respectively (open: adjusted *R*^2^ = 0.42, *F*_33,3472_ = 79.09, *p* < 0.001; closed: adjusted *R*^2^ = 0.13, *F*_28,11,736_ = 64.01, *p* < 0.001) (Appendix S1: Table S5). For the open-canopy vegetation, all variables, their quadratic terms and pairwise interactions were included in the best model except for the N and S deposition interaction. Other than the best models with the lowest AICc, there were four competing models for the open-canopy vegetation and 9 competing models for the closed-canopy vegetation within the delta AICc value <2. These candidate models always contained the N deposition and bedrock and their interaction.

We found that the native to non-native species richness ratio for open-canopy vegetation increases with increasing N deposition, the most for the cation-rich bedrock, but declines the most as well as starts to decrease at the lowest N deposition loads for cation-moderate bedrock (~7 kg N ha^−1^ year^−1^). For the cation-rich and cation-poor bedrock, the critical N deposition is approximately 8–9 kg N ha^−1^ year^−1^. For the closed-canopy vegetation, the shape of the response curve was consistent across bedrock types. Still, for cation-poor bedrock, the decline starts from higher level of N deposition (~12 kg N ha^−1^ year^−1^) in comparison to cation-rich and cation-moderate bedrock (~9–10 kg N ha^−1^ year^−1^) (Figure 4a,b).

Overlay of scaled (scaled to zero mean and unit variance) response curves for species richness and native to non-native species richness ratio (Figure 4c,d) along N deposition gradient showed divergence of curves for closed-canopy vegetation when the N deposition load exceeded 8–11 kg N ha^−1^ year^−1^, suggesting that the non-native species might benefit from higher N deposition loads. For the open-canopy vegetation, the graph created by the same procedure showed a similar pattern but a more apparent divergence of the response curves.

## DISCUSSION

### Nitrogen deposition effect on herbaceous species richness in the United States and CZ

Using the US dataset, we demonstrated that the species richness response to N deposition differs by the bedrock for both vegetation types. The significant influence of N deposition on species richness was previously demonstrated in the United States by Simkin et al. (2016) on the same dataset, but not considering different bedrock types. The inclusion of bedrock and interactions of explanatory variables with the bedrock improved the model *R*^2^ by 16% (from 31%) and 4% (from 13%) in Simkin et al. (2016) for open- and closed-canopy communities, respectively. The N deposition effect on species richness was found to be significant even after controlling for other environmental factors, such as temperature, precipitation, vegetation sampling plot size (sample area), and S deposition. In the Czech dataset, we could not separate the N and S deposition effects, because they both come from the same large emission sources in the CZ; therefore, they had very similar spatial patterns. That congruence is not as strong in the United States due to large agricultural sources in many areas of the country that are not spatially comingled as much as in CZ with industrial sources. Other studies have suggested that climate, landscape context, land use history or bedrock drive species richness patterns in CZ (Divísek & Chytrý, 2018); however, we conclude an effect from combined N and S deposition because this factor was significant even after we included climate and other variables (precipitation and vegetation sampling plot size). The shape of response curves and estimated values for N deposition loads at which the response curves change is comparable in both countries (aside from cation-rich), such that it could be a canonical response to N deposition, not to S deposition, that could be responsible for the species richness changes in the CZ. For the cation-rich communities, the response to N deposition was different, species richness declines across the whole gradient, but we do not have many sites with N deposition loads <10 N ha^−1^ year^−1^.

Plant species richness tended to decline the most on cation-rich bedrock in CZ as hypothesized, though in the United States, the responses were more similar across bedrock types even though overall richness was also higher in the cation-rich sites. Why bedrock type has a more muted effect in the United States would require a more detailed analysis of the vegetation units associated with this substrate and an analysis of the differences in nutrient availability between the types of bedrock categories thus defined. However, in both countries, the results suggest that nitrogen-sensitive communities are associated with bedrock with higher cation content, less so for the cation-poor bedrock.

In both countries, model performance was consistently better for open-canopy than for closed-canopy vegetation. Although explained variability was lower overall in the Czech dataset, the drop was especially pronounced for open-canopy vegetation (47% in the US vs. 12% in the CZ). These differences, especially in the open-canopy vegetation, might arise from different land use legacies not included in our analysis, where the species-rich open vegetation in the CZ is linked to traditional land uses such as grass cutting and grazing (Chytrý et al., 2015). These traditional land uses have long been abandoned, threatening many species. Additionally, species richness in the CZ could also be more influenced by the recovery of soils from soil acidification that happened during the 1970s and 1980s or influenced by opposing processes (eutrophication, oligotrophication) taking place in different vegetation types on the same bedrock, as data from CZ suggest (T. Chuman et al., unpublished manuscript). Thus, these other causes of species richness change might overshadow the N deposition effect itself.

The positive effect of low N deposition on species richness previously reported from the US (Simkin et al., 2016) was observed in both countries, except on cation-rich bedrock in both vegetation types in the Czech dataset. This has not been previously reported in the CZ, and a unimodal relationship further suggests that the RCs represented N deposition. In the cation-rich sites, species richness declined consistently across the entire deposition gradient. Deposition levels at which species richness starts to decrease are well within ranges of the empirical critical loads as defined for the US ecosystems (Pardo et al., 2011, 2019; Simkin et al., 2016; Wilkins et al., 2022) and European ecosystems (Bobbink et al., 2022).

### Ambiguous species richness response to N deposition in the Czech Republic

Contrary to other published studies from Europe, we see a positive effect of N deposition on the species richness not only in the US dataset, as earlier reported by Simkin et al. (2016) but also, albeit modestly, for the Czech dataset at low N deposition loads except for the cation-rich sites. To our knowledge, the positive effect of N deposition at very low N deposition loads on the species richness is rarely reported in other studies from temperate climate regions. The only positive effect of N deposition across the whole gradient was reported by Duprè et al. (2010) on the proportion of grass species or by Bowman et al. (2006) and Spohn et al. (2025) on the species diversity expressed by the Shannon–Wiener index, but it was caused by increased abundance. Simkin et al. (2016) explain the positive effect of increased N availability on species richness at very low deposition loads by the fact that enrichment of N-poor environments leads to a release from N limitation, but not enough to lead to competitive exclusion.

Stevens, Thompson, et al. (2010), when analyzing data from a broad N deposition gradient spanning from 2.4 to 43.5 kg N ha^−1^ year^−1^ in Europe, concluded that the number of species declines across the whole gradient at a greater speed in low levels of N deposition. This is contrary to Simkin et al. (2016) and our results at cation-moderate and cation-poor sites. One possible explanation would be that even though the N deposition in the Stevens, Thompson, et al. (2010) dataset was low, the longer duration of elevated N deposition in Europe might have triggered plant species richness changes at low loads, as the N deposition has a cumulative effect (Clark & Tilman, 2008). On the other hand, even in Stevens, Thompson, et al. (2010), there may simply not be enough data on the low end of the N deposition gradient, as there was only one plot with deposition below 5 kg N ha^−1^ year^−1^, and nearly, all of the plots received 9 kg N ha^−1^ year^−1^ and above. Thus, even with moderate variation from other factors, an unimodal relationship could not be detected. In the United States, there are still many remote areas where the anthropogenically elevated atmospheric N deposition is below 2–3 kg ha^−1^ year^−1^. This might explain the intercontinental differences between Stevens, Thompson, et al. (2010) and our work; however, the unimodal pattern was detected even in the Czech dataset, where the species richness responded positively to N (and S) deposition and started to decrease above average N deposition loads (~10–14 kg ha^−1^ year^−1^) except for the cation-rich bedrock, where species richness decreases across the entire N deposition gradient. The European study of Stevens, Thompson, et al. (2010) focused only on dry acid grasslands and different habitats analyzed altogether in our study might show slightly shifted response to N deposition. However, on cation-rich bedrock, the response of species richness to N deposition in the Czech dataset aligns with previous results.

### Species richness as an indicator of N deposition effect

Species richness is a rather coarse indicator of the N deposition impact because numerous studies have shown that weak plant competitors are often outcompeted by other plants, such as those favoring N or invasive species, without affecting the total number of species (Bobbink et al., 1998). Hillebrand et al. (2018) also suggest that species turnover indices, rather than species richness alone, provide deeper insight into temporal biodiversity trends by revealing how community composition and species dominance change over time. Our analysis shows that species richness changes as a result of N deposition, as does the ratio between native and non-native species. Above a specific N deposition load, the overlayed curves diverge, and when both start declining, the native to non-native species richness ratio declines at a faster rate than species richness. Based on the results of Brooks (2003), Chen et al. (2025), Pyšek and Lepš (1991) and Valliere et al. (2022), which show that the number of non-native species increases with N deposition, we might speculate that the non-native species may benefit more from N deposition or that non-native species decline more slowly than native species. Based on this assumption, it appears that in closed-canopy vegetation, non-native species benefit from N deposition across all bedrock types exceeding 9–12 kg N ha^−1^ year^−1^ (Figure 4). Open-canopy vegetation seems to be less resistant to plant invasion as the divergence of curves is more pronounced. We expect that there would be high variability within these broad vegetation types as the level of non-native species presence significantly differs by vegetation types at much lower classification levels (Chytrý, Jarošík, et al., 2008; Chytrý, Maskell, et al., 2008; Pyšek et al., 2012). We did not perform the analysis for the Czech dataset, as the data for the native to non-native species were not available, but we expect the response to be less significant, as the number of non-native species is relatively low per site in the Czech flora (Divíšek & Chytrý, 2018). These authors report the mean species richness of non-native species to be 0.53 per plot (based on 18,012 grassland plots and 13,051 forest plots) compared with 21.9 native species and the modeled number of non-native species to be also rather low, with up to five species across the country. The levels of non-native species presence are also generally higher in the US than in Europe as a larger proportion of alien species was provided by European habitats for invasion to North America than vice versa (Kalusová et al., 2015). The increase of non-native species due to N deposition, however, could be the reason why we see some increase in species richness in the Czech dataset, whereas Stevens, Thompson, et al. (2010) presented a decrease in species richness along the entire N deposition gradient in Europe. The presence of non-native species in dry acid grasslands analyzed by Stevens, Thompson, et al. (2010) is low (Chytrý, Jarošík, et al., 2008), as this type of vegetation with limited resource availability tends to be more resistant to invasion (Pyšek et al., 2012).

### Bedrock influence on plant species richness response to N deposition

Although the defined bedrock types are not directly linked to soil properties, these broad categories reflect several soil characteristics, such as pH, potential soil buffering capacity, and nutrient balances. There are several studies assessing the pH effect on plant species richness and concluding that acidification reduces the richness, most likely due to aluminum toxicity (Stevens, Duprè, et al., 2010) or/and due to a shift in nutrient ratios (Braakhekke & Hooftman, 1999; Roem & Berendse, 2000). Out of three bed-rock types, the cation-poor bedrock often has the least balanced nutrient ratios and probably multiple nutrient limitations compared to cation-rich bedrock. If an excess of deposited N contributes to soil acidification, cation-poor bedrock should also be the most vulnerable to aluminum toxicity, and a decline in species richness would occur. Contrastingly, cation-rich bedrock is resistant to acidification. However, the increase in strong competitors might be P-limited on carbonaceous rocks (Carroll et al., 2003), which are included in the cation-rich bedrock type, as soil carbonates suppress P availability (Sposito, 2008).

Therefore, we hypothesized that N deposition would lead to a greater response of species richness on cation-rich bedrock, and our results somewhat align with this assumption, more so in the CZ than US data, showing a steeper decline in species richness on cation-rich sites in comparison to cation-poor sites, also in line with theory (Harpole & Tilman, 2007). Sites on the cation-moderate bedrock behaved differently in both countries, being more similar to cation-rich sites in the United States and cation-poor sites in the CZ.

Closed-canopy vegetation showed a steep decline in species richness in the CZ for cation-rich sites, but there was a unimodal response in the United States. The divergence from theory might arise from lower N deposition in the United States or the different resistance to plant invasion, as sites on cation-rich and cation-moderate bed-rock types tend to be more prone to plant invasion, as our analysis of the US data suggests. Thus, outcompeted native species might be balanced by invasive species, and species richness change is less pronounced. In CZ, a decline in species richness in closed-canopy vegetation on cation-rich sites across the whole gradient could also represent the interplay between N deposition effect on tree species vegetation cover that would limit light to the understorey vegetation. This is the most important driver of species composition changes as suggested in the latest study of Landuyt et al. (2024). In addition, an increase in canopy cover has been documented in many forests in CZ (Chudomelová et al., 2017; Vild et al., 2024).

Simkin et al. (2016) showed that species richness tended to decrease with N deposition at lower levels under more acidic conditions (lower pH). Given the results of Simkin et al. (2016), we expected to see a higher sensitivity of species richness to N deposition on cation-poor bedrock. However, we did not find differences in the N deposition levels, but we found differences in the magnitude of changes. Sites on cation-poor bedrock generally have fewer species; thus, there are fewer species to lose in open-canopy sites. In closed-canopy vegetation, the cation-poor bedrock sites tend to be similarly responsive to N-induced species richness changes but with lower richness. The results suggest that the bedrock types cannot be directly associated with pH, and it captures different phenomena. This is consistent with the observation of a wide variation of soil pH within the same bedrock type, as shown in the boxplot (Appendix S1: Figure S6). Regardless, we found that bedrock is a strong predictor that consistently controls species response patterns to N deposition for both countries and vegetation types, and nutrient ratios probably influence the response of plant species richness to nitrogen deposition.

The bedrock types, as derived from the GLiM and the MGeoReaCR, suggested to be a valuable proxy variable for soil properties over a broad geographical scale, yielding comparable ranges of critical loads for nitrogen deposition in both countries. Bedrock might be a more robust predictor than modeled soil chemical properties if these are not available from on-site soil sampling laboratory analyses, as pH shows very high fine-scale spatial variability due to microtopography or vegetation (Garten et al., 2007; Jackson & Caldwell, 1993; Šamonil et al., 2011). Bedrock has also been identified as an important factor when assessing forest N dynamics (Williard et al., 2005). Where available, however, either variable has been found to be predictive of the effects of N deposition, though it appears they are capturing different phenomena.

In addition, the influence of the bedrock may be overshadowed by conflicting processes occurring in different types of vegetation. Chuman et al. (in prep.) found that despite similar N deposition loads, pine forests, unlike oak and beech forests, were experiencing oligotrophication (loss of eutrophic species) instead of eutrophication (increase in eutrophic species) on the same cation-poor bedrock. The opposite processes in different vegetation types might be the reason for a relatively weak N deposition–bedrock interaction.

The influence of bedrock may also be masked by chronically high N deposition. Studies by Dirnböck et al. (2014), Gilliam and Hockenberry (2006) or Schleppi et al. (1999) did not find the response of species richness to N deposition or addition, probably also due to chronically high ambient N deposition, which might have shifted the species composition to more N demanding species sometime in the past.

However, the significance of bedrock supports the general understanding that bedrock differences control the soil properties, including the soil buffering capacity, and thus, resistance to soil acidification is influenced by bedrock. Soil pH, on the other hand, is a surficial measurement. Thus, although it may better align with what the plant roots are experiencing, it is much more dynamic in space and time and can be greatly affected by plant root exudates and decomposition rates, among other factors that operate at much finer spatial scales.

Looking ahead, bedrock influence on soil properties is likely to shift under future climate conditions. Bedrock controls the buffering capacity and nutrient release, but these are also climate-dependent. With warming, increased weathering rates, altered soil moisture regimes, and changes in organic matter decomposition may either amplify or diminish bedrock-derived differences among sites. Thus, the relative importance of bedrock may become more dynamic in the future, with climate-driven shifts in soil pH, nutrient availability, and biological activity reshaping how lithology constrains vegetation.

### Is it only the N deposition which is driving the changes?

The species richness changes in this study are attributed to N deposition; however, other studies stress a more important role played by light availability, forest management, or herbivory rather than as a direct effect of increasing N availability (Landuyt et al., 2024; Perring et al., 2018; Roth, Michiels, Puhlmann, Sucker, & Hauck, 2021; Roth, Michiels, Puhlmann, Sucker, Winter, & Hauck, 2021; Verheyen et al., 2012; Vild et al., 2024). The reason why a decrease in light availability better explained vegetation changes compared with N deposition under controlled experiments might be attributed to the indirect N deposition effect on aboveground biomass. Braun et al. (2010), Etzold et al. (2020) and Forsmark et al. (2020) found increased forest productivity along N deposition gradients, which means increased canopy closure of the tree layer and decreased light availability to the herb layer.

We do not have data to assess these other relevant factors. We also do not know the N deposition legacy at each site prior to sampling, which is also important (Bobbink et al., 2022; Clark & Tilman, 2008), nor is there detailed spatial data for light availability at the canopy floor for either country for the years covered with these datasets.

## CONCLUSIONS

Our analysis shows the effect of N deposition on species richness across broad environmental gradients for open- and closed-canopy vegetation in the United States and the CZ. This comparison helps us understand how species richness changes across a much broader N deposition gradient, using the same explanatory variables and statistical approach, especially in low nitrogen deposition loads, data that are lacking in most of Europe. The species richness response to increasing N deposition differed by the bedrock, especially in CZ and less so in the United States, and vegetation type. In the United States, N deposition is positively correlated to species richness at very low deposition loads, then becomes negatively correlated when N deposition reaches approximately 10–14 kg N ha^−1^ year^−1^ for closed and open canopies. We also showed with US data that the native to non-native species richness ratio changes with N deposition. In the CZ, species richness on both cation-moderate and cation-poor bedrock exhibits a weak initial increase with rising N deposition until reaching 10–13 kg N ha^−1^ year^−1^, after which it declines— a pattern not previously documented for this region. On cation-rich bedrock, species richness declines across the entire gradient. Our results overall are in general agreement with the empirical critical loads as defined for the US ecosystems by Pardo et al. (2011, 2019), Simkin et al. (2016), Wilkins et al. (2022) and European ecosystems by Bobbink et al. (2022) (Appendix S1: Table S6) and the bedrock GLiM database suggested to be a valuable dataset if pH is not readily available. Generally, sites on cation-rich bedrocks exhibit higher species richness and a steeper decline due to N deposition. Therefore, to prevent a decline in species diversity, it is essential to focus on protecting these sites.

## Supplementary Material

Supplement1

SUPPORTING INFORMATION

Additional supporting information can be found online in the Supporting Information section at the end of this article.

## Figures and Tables

**TABLE 1:** Explanatory variables and associated datasets used in this study.

Variable	United States	Czech Republic

Bedrock	Global Lithological Map Database (GLiM) (Hartmann & Moosdorf, 2012)	Map of Geochemical reactivity of rocks of the Czech Republic (Chuman et al., 2014)
N deposition	Long-term wet deposition (NADP-Ndep) plus NADP-Ndep × dry: wet modeled (CMAQ) ratio (Simkin et al., 2016)	Modeled wet nitrogen deposition (Oulehle et al., 2016) × dry: wet ratio derived from the EMEP database
Precipitation	30-year precipitation normal (PRISM, 1981–2010) (Simkin et al., 2016)	Average from 1990 to 2013, (Czech Hydrometeorological Institute, 2024)
Temperature	30-year temperature normal (PRISM, 1981–2010) (Simkin et al., 2016)	Average from 1990 to 2013, (Czech Hydrometeorological Institute, 2024)
S deposition	Total sulfur deposition (wet and dry deposition from CMAQ model) (Simkin et al., 2016)	Modeled total sulfur deposition (Oulehle et al., 2016)
Vegetation type	National Land Cover Database in the U.S. (Simkin et al., 2016)	Adopted from (Simkin et al., 2016)

**TABLE 2:** The classification of the GLiM classes to cation levels, as used in this study for the United States.

GLiM code	GLiM class	Typical rock representative

Cation-rich		
py	Pyroclastics	Sediments of volcanic origin. Typical pyroclastics are tuff, volcanic breccias, or ash
mt_pu	Metamorphics – pure carbonate	Marble
sm	Mixed sedimentary rocks	All sediments where carbonate is mentioned but not dominant, interlayered sandstone and limestone, shaley marl
sc	Carbonate sedimentary rocks	Dominated by carbonate rocks
Cation-moderate		
va	Acid volcanic rocks	Typically rhyolites, trachytes, or dacites
vi	Intermediate volcanic rocks	Typically andesites
vb	Basic volcanic rocks	Basalt-type rocks
pa	Acid plutonic rocks	Represent plutonic rocks containing quartz, granites, and their relatives, also quartzdiorites and quartz-monzonites
pb	Basic plutonic rocks	Plutonic rocks rich in mafic minerals, like gabbro and peridotite
pi	Intermediate plutonic rocks	Dominated by diorite, monzonite, syenite and their subtypes
mt (except for mt_pu)	Metamorphics	Typically shales, gneiss
Cation-poor		
su	Unconsolidated sediments	Usually of Cenozoic age dune sands, alluvial deposits, loess, sands, should be without carbonates
ss	Siliciclastic sedimentary rocks	Sandstone, mudstone, and greywacke, siliciclastic sedimentary rocks are without mapped carbonate influence

*Note*: The metamorphics at the first level classification had to be divided into carbonaceous and non-carbonaceous metamorphics based on the information in the second-level classification of the GLiM database. None of the sites were found on the evaporites.

**TABLE 3:** Pearson correlation coefficients among explanatory variables for the US and the Czech datasets.

Variable	Precipitation	Temperature	N dep.	S dep.

USA				
Precipitation				
Temperature	0.446			
Nitrogen deposition	0.445	0.549		
Sulfur deposition	0.652	0.549	0.704	
CZ				
Precipitation				
Temperature	−0.847			
Nitrogen deposition	0.794	−0.682		
Sulfur deposition	0.423	−0.336	0.766	

**TABLE 4:** Descriptive statistics of the environmental variables for both countries showing the general span of environmental gradients.

	Nitrogen deposition (kg ha^−1^ year^−1^)		Temperature (°C)		Precipitation (mm)		Sulfur deposition (kg ha^−1^ year^−1^)	
	CZ	USA	CZ	USA	CZ	USA	CZ	USA
Statistic	1990–2013	2000–2012	1990–2013	1981–2010	1990–2013	1981–2010	1990–2013	2000–2012

Mean	13.3	7.8	8.3	8.8	766	991	15.6	7.8
Median	12.3	8.8	8.5	8.3	694	1046	11.0	6.9
SD	3.3	3.8	1.3	4.7	222	386	12.9	6.2
Min.	7.8	1.0	2.2	−1.7	442	100	3.8	0.3
Max.	24.9	20.2	10.8	22.8	1583	5008	58.0	43.0

*Note*: For span of environmental gradients by vegetation type and bedrock see Appendix S1: Table S1.

**TABLE 5:** Descriptive statistics of the species richness per plot in the Czech and the US datasets for all bedrock types and by bedrocktypes.

Country	VegSimkin	Bedrock	Count	Mean	Median	SD	Min.	Max.

CZ	Closed		9569	17	10	16.0	1	83
		Cation-rich	1915	21	10	20.0	1	83
		Cation-moderate	4984	17	10	15.0	1	64
		Cation-poor	2670	17	10	15.0	1	70
CZ	Open		12,932	24	12	23.0	1	127
		Cation-rich	3616	29	14	28.0	1	93
		Cation-moderate	5692	22	10	21.0	1	64
		Cation-poor	3624	22	11	22.0	1	127
US	Closed		11,765	18	16	12.8	1	85
		Cation-rich	1294	25	24	13.2	1	73
		Cation-moderate	5651	18	16	12.1	1	79
		Cation-poor	4820	17	14	12.9	1	85
US	Open		3506	18	16	12.7	1	83
		Cation-rich	579	24	24	10.8	1	73
		Cation-moderate	1265	18	14	12.7	1	74
		Cation-poor	1662	17	14	12.8	1	83

*Note*: “Closed” and “open” denote closed- or open-canopy vegetation types.

## Data Availability

No new data were collected for this study. Existing datasets used are listed in Table 1.
