# Supplementary material for: Plant species richness response to atmospheric nitrogen deposition across bedrock types in the United States and Czechia
Source: Ecosphere. Author manuscript; Available in PMC 2026 Aug 1. (PMC13426850; doi:10.1002/ecs2.70663)
Supplement: Supplement1 [file NIHMS2181410-supplement-Supplement1.pdf]

Appendix S1

Journal name: **Ecosphere**

Title: **Plant species richness response to atmospheric nitrogen deposition across bedrock types in the United States and Czechia**

Authors: **Tomas Chuman**, **Christopher M. Clark**

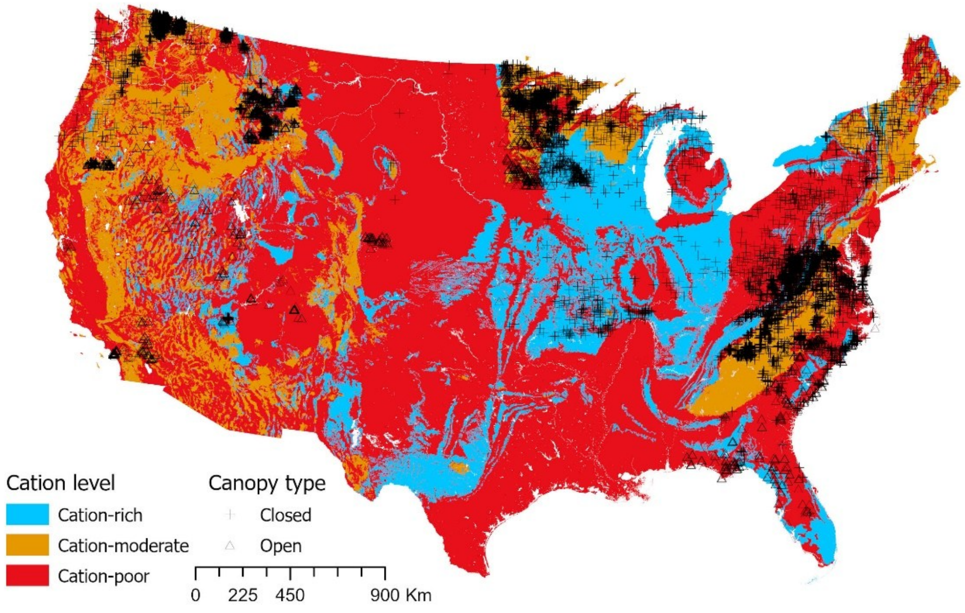

Fig. S1. Spatial distribution of bedrock Cation levels used in this study, based on the GLiM database (Hartmann and Moosdorf 2012), and vegetation samples in the US.

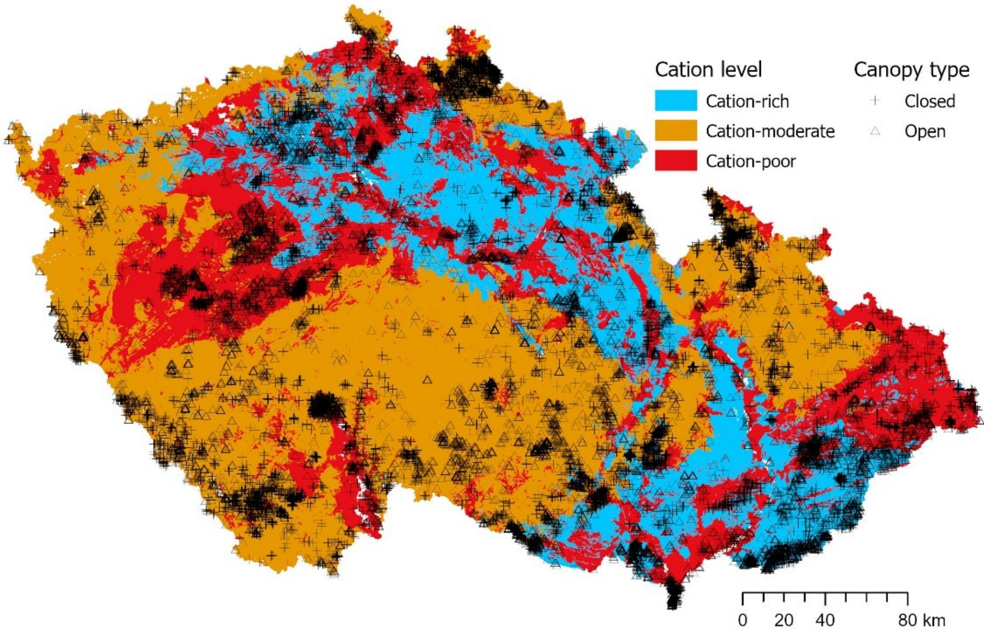

Fig. S2. Spatial distribution of bedrock Cation levels used in this study, based on the MGeoReaCR database (Chuman et al. 2014), and vegetation samples in Czechia.

Table S1. Descriptive statistics for the environmental variables by country, vegetation type and bedrock level, showing the number of vegetation samples (n) and mean, standard deviation (sd), median, minimum (min) and maximum (max) value of each environmental variable.

Variable	Country	Vegetation type	Bedrock	n	mean	sd	median	min	max
N deposition [kg.ha^{-1}.yr^{-1}]	CZ	Closed	Cation-rich	1915	13.7	2.5	13.6	7.9	22.6
			Cation-moderate	4984	15.1	4.2	13.7	8.9	24.9
			Cation-poor	2670	14.2	3	13.3	8.7	22.4
		Open	Cation-rich	3616	11.6	1.8	11.6	7.9	18.8
			Cation-moderate	5692	13	3.3	11.9	7.9	23
			Cation-poor	3624	12	2.3	11.4	7.8	20.3
	US	Closed	Cation-rich	1294	9.5	3.6	10	1.6	16.9
			Cation-moderate	5651	7.5	3.5	8.1	1.2	17.2
			Cation-poor	4820	8.9	3.2	9.8	1.3	20.2
		Open	Cation-rich	579	7	4.6	4.3	1.1	16.4
			Cation-moderate	1265	5	3.9	2.9	1	16.2
			Cation-poor	1662	7.1	4.5	6.6	1.1	19.4
Precipitation [mm]	CZ	Closed	Cation-rich	1915	714.4	148	660.9	466.6	1258.3
			Cation-moderate	4984	844.1	250.6	776.1	493.4	1562.7
			Cation-poor	2670	757.9	230.9	682.5	487.3	1455.9
		Open	Cation-rich	3616	688.5	149	652.6	454	1249.9
			Cation-moderate	5692	817.8	237.6	759.2	485.7	1583.5
			Cation-poor	3624	685.6	175.1	631.2	441.6	1409.5
	US	Closed	Cation-rich	1294	973.4	241.1	970.9	376.4	2117.6
			Cation-moderate	5651	1064.9	435.6	1084.3	324.6	5007.5
			Cation-poor	4820	1103.9	279.6	1133.3	304.7	4346
		Open	Cation-rich	579	785.3	276.1	771.9	221.5	1491.4
			Cation-moderate	1265	637.6	258.9	600.5	107.7	2086.1
			Cation-poor	1662	766.7	397	711.1	99.9	1697.3
Temperature [°C]	CZ	Closed	Cation-rich	1915	8.6	0.8	8.8	5.4	10.3
			Cation-moderate	4984	7.7	1.5	7.9	2.6	10.3
			Cation-poor	2670	8.5	1.1	8.7	3.7	10.6
		Open	Cation-rich	3616	8.9	0.8	9	6.3	10.8
			Cation-moderate	5692	7.8	1.3	8	2.2	10.4
			Cation-poor	3624	8.9	0.9	8.9	2.5	10.8
	US	Closed	Cation-rich	1294	8.8	4	7.9	-1.5	19.7
			Cation-moderate	5651	8.1	4.3	7.6	-1.4	17.5
			Cation-poor	4820	9.9	4.2	10.2	0.2	22.6
		Open	Cation-rich	579	7.1	4.9	6.4	-0.9	21.3
			Cation-moderate	1265	6	3.8	4.9	-1.7	20.8
			Cation-poor	1662	10.6	6.2	8.9	0.4	22.8
S deposition [kg.ha^{-1}.yr^{-1}]	CZ	Closed	Cation-rich	1915	25.6	8.9	23.4	10.2	56.4
			Cation-moderate	4984	27.3	15.6	19	8.2	58
			Cation-poor	2670	26	10.1	24.3	9.3	57.3
		Open	Cation-rich	3616	7.1	1.7	7	3.8	13.2
			Cation-moderate	5692	7.9	3.5	6.4	3.8	18.1
			Cation-poor	3624	7.1	2.3	6.3	3.8	16
	US	Closed	Cation-rich	1294	8.2	5.1	7.3	0.7	30.3
			Cation-moderate	5651	7.3	5.2	6.8	0.4	20.3
			Cation-poor	4820	11.3	6.8	11.9	0.5	43
		Open	Cation-rich	579	4	3.6	1.7	0.4	17.9
			Cation-moderate	1265	2.4	2.9	1.2	0.3	15.6
			Cation-poor	1662	4.2	3.8	2.8	0.4	25.5

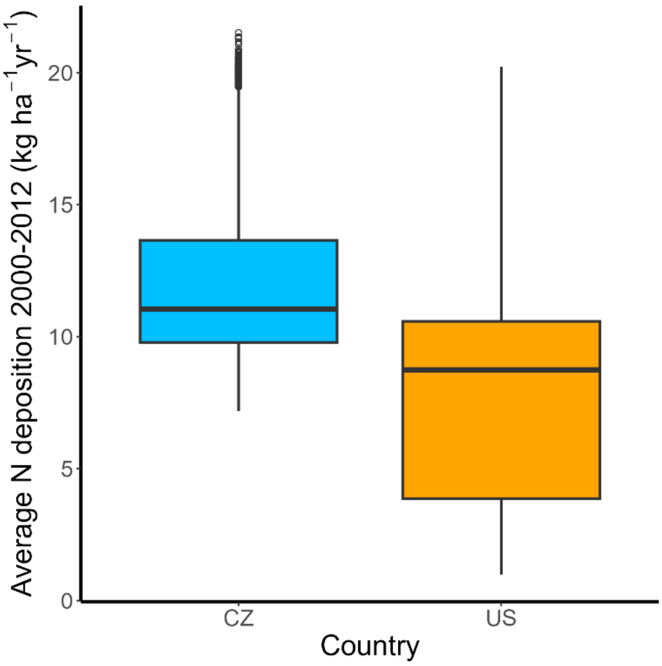

Fig. S3. Comparison of the Average Nitrogen Deposition in kg ha^{-1} yr^{-1} for the Czech Republic (CZ) and the United States (US). The boxplots show significant differences between these two countries and compare the same period 2000-2012. The box shows the interquartile range (IQR) (25th-75th percentile) with the median. Whiskers extend to values within 1.5 x IQR, and data points beyond are shown as outliers.

Table S2. The best model and competing models (all with ΔAIC < 2) for the United States dataset. Regression coefficients, adjusted R^2 and AICc for the best models of species richness response to N deposition for the US open- and closed-canopy vegetation datasets. A "+" and "-" sign indicates which categorical variable or interaction with a categorical variable was included in the model and which variable was not. Delta AICc shows the difference between the best model and the null model or the best model and the competing model AICc. The red signs in the cells show the differences in included terms from the selected model.

	open canopy						
	selected	competing models					
**Intercept**	-6.29	-0.95	-2.87	-0.65	-2.66	-6.47	-8.94
**Bedrock**	+	+	+	+	+	+	+
**N dep.**	2.83	2.29	2.71	2.24	2.62	2.82	3.27
**N dep.^{2}**	-0.12	-0.09	-0.11	-0.08	-0.09	-0.12	-0.13
**Precip.**	0.04	0.04	0.04	0.04	0.05	0.04	0.05
**Precip.^{2}**	0.00	0.00	0.00	0.00	0.00	0.00	0.00
**S dep.**	1.99	2.48	1.76	2.51	1.68	1.98	1.05
**S dep.^{2}**	0.04	0.04	0.10	-	-	-	-
**Sample Area**	0.01	0.01	0.01	0.01	0.01	0.01	0.01
**Sample Area^2**	0.00	0.00	0.00	0.00	0.00	0.00	0.00
**Temp.**	0.15	-0.55	-0.42	-0.65	-0.65	0.12	0.16
**Temp.^{2}**	-0.03	0.01	0.01	0.02	0.02	-0.02	-0.02
**Bedrock : N dep.**	+	+	+	+	+	+	+
**Bedrock : N dep.^{2}**	+	+	+	+	+	+	+
**Bedrock : Precip.**	+	+	+	+	+	+	+
**Bedrock : Precip.^{2}**	+	+	+	+	+	+	+
**Bedrock : S dep.**	-	+	+	+	+	-	-
**Bedrock : S dep.^{2}**	-	-	+	-	-	-	-
**Bedrock : Temp.**	+	+	+	+	+	+	+
**Bedrock : Temp.^{2}**	+	+	+	+	+	+	+
**N dep. : Precip.**	0.00	0.00	0.00	0.00	0.00	0.00	0.00
**N dep. : S dep.**	-0.17	-0.17	-0.19	-0.13	-0.12	-0.13	-0.11
**N dep. : Temp.**	-0.14	-0.14	-0.13	-0.14	-0.14	-0.14	-0.14
**Precip. : Temp.**	0.00	0.00	0.00	0.00	0.00	0.00	0.00
**S dep. : Precip.**	0.00	0.00	0.00	0.00	-	0.00	-
**S dep. : Temp.**	-0.09	-0.09	-0.10	-0.09	-0.10	-0.09	-0.10
**df**	32.00	34.00	36.00	33.00	32.00	31.00	30.00
**logLik**	-12770.45	-12768.47	-12766.45	-12769.90	-12770.96	-12772.16	-12773.42
**AICc**	25605.51	25605.62	25605.68	25606.45	25606.54	25606.89	25607.38
**Adj. R^2**	0.47						
**null model AICc**	27786.52						
**ΔAICc to null m.**	2181.02						
**ΔAICc to selected**		0.12	0.17	0.94	1.03	1.38	1.87

	closed canopy			
	selected	competing models		
**Intercept**	15.14	15.61	16.06	15.22
**Bedrock**	+	+	+	+
**N dep.**	4.07	4.01	4.05	4.43
**N dep.^{2}**	-0.16	-0.16	-0.15	-0.18
**Precip.**	-0.03	-0.03	-0.03	-0.03
**Precip.^{2}**	0.00	0.00	0.00	0.00
**S dep.**	-0.64	-0.60	-0.54	-0.65
**S dep.^{2}**	0.03	0.03	0.03	0.03
**Sample Area**	0.01	0.01	0.01	0.01
**Sample Area^2**	0.00	0.00	0.00	0.00
**Temp.**	0.24	0.26	0.08	0.18
**Temp.^{2}**	-0.02	-0.02	-	-0.02
**Bedrock : N dep.**	+	+	+	+
**Bedrock : N dep.^{2}**	-	-	-	+
**Bedrock : Precip.**	+	+	+	+
**Bedrock : Precip.^{2}**	+	+	+	+
**Bedrock : S dep.**	+	+	+	+
**Bedrock : S dep.^{2}**	-	-	-	-
**Bedrock : Temp.**	+	+	+	+
**Bedrock : Temp.^{2}**	-	-	-	-
**N dep. : Precip.**	-	0.00	-	-
**N dep. : S dep.**	-	-	-	-
**N dep. : Temp.**	-0.07	-0.08	-0.09	-0.07
**Precip. : Temp.**	0.00	0.00	0.00	0.00
**S dep. : Precip.**	0.00	0.00	0.00	0.00
**S dep. : Temp.**	0.05	0.06	0.05	0.05
**df**	28.00	29.00	27.00	30.00
**logLik**	-45573.90	-45573.54	-45575.80	-45572.81
**AICc**	91203.95	91205.22	91205.72	91205.77
**Adj. R^2**	0.17			
**null model AICc**	93414.60			
**ΔAICc to null m.**	2210.66			
**ΔAICc to selected**		1.27	1.78	1.83

Table S3. PCA loadings of correlated environmental variables and their varimax-rotated loadings for the Czech Republic datasets for each principal component (PC) and rotated component (RC).

	open canopy vegetation				closed canopy vegetation			
**PCA loadings**	**PC1**	**PC2**	**PC3**	**PC4**	**PC1**	**PC2**	**PC3**	**PC4**
Precipitation	0.94	-0.19	0.28	0.02	0.92	-0.29	0.27	-0.04
Temperature	-0.86	0.48	0.18	0.03	-0.82	0.52	0.23	0.02
Nitrogen deposition	0.95	0.27	-0.11	0.12	0.97	0.23	0.00	0.12
Sulphur deposition	0.93	0.36	-0.01	-0.12	0.8	0.59	-0.07	-0.08
Proportion of explained variability	0.84	0.12	0.03	0.01	0.77	0.19	0.03	0.01
Cumulative proportion of explained variability	0.84	0.96	0.99	1.00	0.77	0.96	0.99	1
**Loadings after Varimax rotation**	**RC1**	**RC2**	**RC3**	**RC4**	**RC1**	**RC2**	**RC3**	**RC4**
Precipitation	0.49	0.56	0.67	0.01	0.68	0.36	0.02	0.64
Temperature	-0.33	-0.91	-0.26	-0.01	-0.24	-0.21	-0.02	-0.95
Nitrogen deposition	0.88	0.38	0.25	0.14	0.34	0.81	0.16	0.44
Sulphur deposition	0.91	0.28	0.29	-0.11	0.15	0.98	-0.04	0.14

Table S4. The best model and competing models (ΔAIC < 2) for the Czech dataset (there were no competing models for the open canopy community). Regression coefficients, adjusted R^2 and AICc for the best models of species richness response to N deposition for Czech open- and closed-canopy vegetation datasets. A "+" and "-" sign indicates which categorical variable or interaction with a categorical variable was included in the model and which variable was not. RC stands for varimax rotated components, providing information in brackets, which environmental gradient it represents. Delta AICc shows the difference between the best model and the null model or the best model and the competing model AICc. The red signs in the cells show the differences in included terms from the selected model.

**open canopy**		**closed canopy**			
	selected		selected	competing models	
**Intercept**	30.60	**Intercept**	18.71	18.58	18.71
**Bedrock**	+	**Bedrock**	+	+	+
**RC1 (NS dep.)**	-4.50	**RC1 (Precip.)**	-1.27	-1.29	-1.25
**1 (NS dep.)^2**	-0.28	**RC1 (Precip.)^2**	1.05	1.05	0.99
**RC2 (Temp.)**	2.40	**RC2 (NS dep.)**	-3.11	-3.12	-3.12
**2 (Temp.)^2**	-1.44	**RC2 (NS dep.)^2**	1.21	1.20	1.15
**Sample Area**	0.02	**Sample Area**	0.00	0.00	0.00
**mple Area^2**	0.00	**Sample Area^2**	-	0.00	-
**Bedrock : RC1 (NS dep.)**	+	**Bedrock : RC1 (Precip.)**	+	+	+
**: RC1 (NS dep.)^2**	+	**Bedrock : RC1 (Precip.)^2**	+	+	+
**Bedrock : RC2 (Temp.)**	+	**Bedrock : RC2 (NS dep.)**	+	+	+
**k : RC2 (Temp.)^2**	+	**Bedrock : RC2 (NS dep.)^2**	+	+	+
**RC1 (NS dep.) : RC2 (Temp.)**	-0.23	**RC1 (Precip.) : RC2 (NS dep.)**	0.23	0.23	-
**df**	19.00	**df**	18.00	19.00	17.00
**logLik**	-49957.08	**logLik**	-35557.98	-35557.52	-35559.94
**AICc**	99952.21	**AICc**	71152.03	71153.12	71153.94
**Adj. R^2**	0.12	**Adj. R^2**	0.07		
**null model AICc**	101577.06	**null model AICc**	71826.47		
**ΔAICc to null m.**	1624.85	**ΔAICc to null m.**	674.43		
**ΔAICc to selected**		**ΔAICc to selected**		1.08	1.91

Fig. S4. Relationship between average nitrogen deposition and average sulphur deposition and the first rotated component (RC1) for the open canopy vegetation in Czechia.

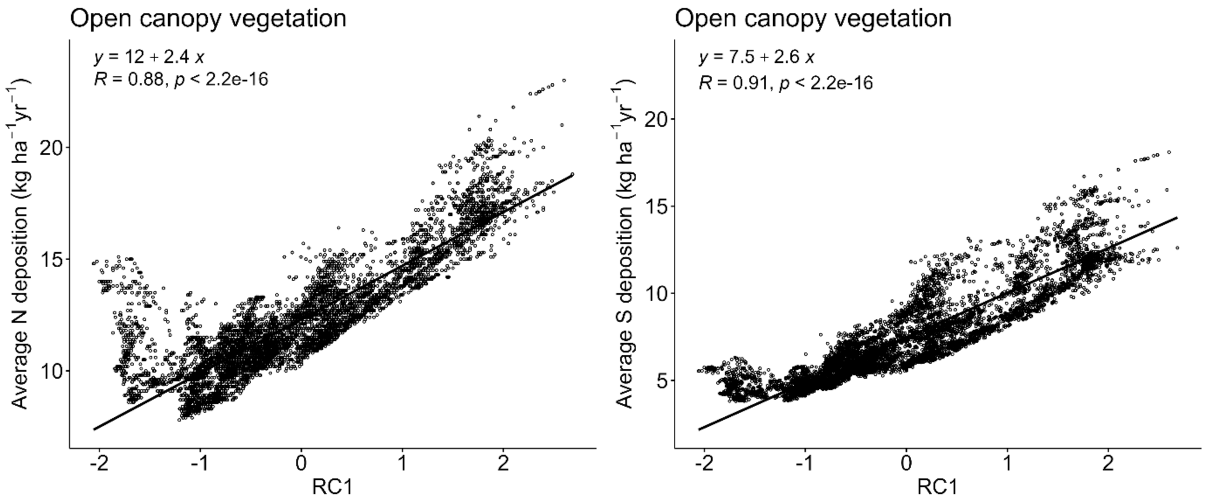

Fig. S5. Relationship between average nitrogen deposition and average sulphur deposition and the second rotated component (RC2) for the closed canopy vegetation in Czechia.

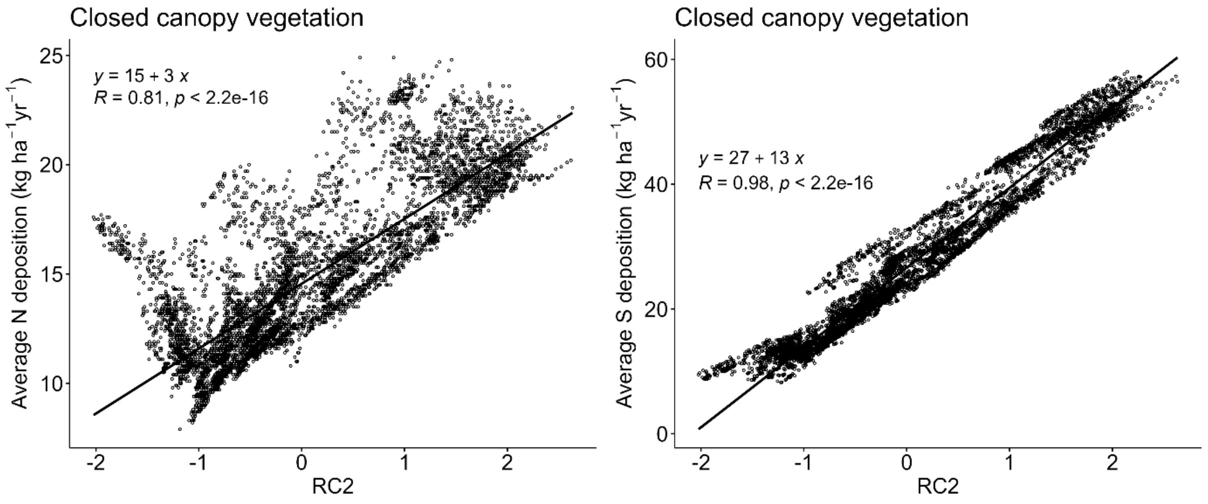

Table S5. The best model and the competing models (ΔAIC < 2) for the native to non-native species richness ratio in the US dataset. The regression coefficients, AICc and adjusted R^2 for the best-tested models for the US native to non-native species richness ratio response to N deposition for the open and closed canopy vegetation datasets. A “+” and “-” sign indicates which categorical variable or interaction with a categorical variable was included in the model and which variable was not. RC stands for varimax rotated components, providing information in brackets, which environmental gradient it represents. Delta AICc shows the difference between the best model and the competing model AICc. The red signs in the cells show the differences in included terms from the selected model.

	open canopy						closed canopy									
	selected	competing models					selected	competing models								
**Intercept**	-6.85	3.14	2.29	-5.23	-5.82	**Intercept**	-20.24	-20.91	-19.55	-20.34	-19.89	-21.04	-19.26	-20.09	-20.56	-19.92
**Bedrock**	+	+	+	+	+	**Bedrock**	+	+	+	+	+	+	+	+	+	+
**N dep.**	3.08	2.63	3.26	2.07	2.78	**N dep.**	4.54	4.56	4.30	4.54	4.44	4.57	4.26	4.31	4.47	4.42
**N dep.^{2}**	-0.18	-0.18	-0.19	-0.13	-0.18	**N dep.^{2}**	-0.13	-0.13	-0.13	-0.12	-0.13	-0.13	-0.14	-0.13	-0.13	-0.13
**Precip.**	0.01	-0.01	-0.01	0.01	0.01	**Precip.**	0.02	0.02	0.02	0.02	0.02	0.02	0.02	0.02	0.02	0.02
**Precip.^{2}**	0.00	-	-	0.00	0.00	**Precip.^{2}**	0.00	0.00	0.00	0.00	0.00	0.00	0.00	0.00	0.00	0.00
**S dep.**	0.57	2.13	1.10	1.01	1.12	**S dep.**	-1.07	-1.15	-0.98	-1.19	-0.94	-1.28	-0.76	-1.05	-1.04	-1.05
**S dep.^{2}**	0.17	0.15	0.15	0.15	0.17	**S dep.^{2}**	0.07	0.07	0.07	0.07	0.07	0.07	0.08	0.08	0.07	0.07
**Sample Area**	0.01	0.01	0.01	0.01	0.01	**Sample Area**	0.02	0.02	0.02	0.02	0.02	0.02	0.02	0.02	0.02	0.02
**Sample Area^2**	0.00	0.00	0.00	0.00	0.00	**Sample Area^2**	0.00	0.00	0.00	0.00	0.00	0.00	0.00	0.00	0.00	0.00
**Temp.**	-0.23	-0.66	-0.67	-0.29	-0.28	**Temp.**	0.33	0.43	0.42	0.40	0.30	0.52	0.32	0.53	0.40	0.39
**Temp.2**	0.05	0.06	0.06	0.06	0.05	**Temp.^{2}**	-	**-0.01**	-	-	-	**-0.01**	-	**-0.01**	**-0.01**	-
**Bedrock : N dep.**	+	+	+	+	+	**Bedrock : N dep.**	+	+	+	+	+	+	+	+	+	+
**Bedrock : N dep.^{2}**	+	+	+	-	+	**Bedrock : N dep.^{2}**	-	-	-	-	-	-	-	-	-	-
**Bedrock : Precip.**	+	-	-	+	+	**Bedrock : Precip.**	+	+	+	+	+	+	+	+	+	+
**Bedrock : Precip.^{2}**	+	-	-	+	+	**Bedrock : Precip.^{2}**	-	-	-	-	-	-	-	-	-	-
**Bedrock : S dep.**	+	+	+	+	+	**Bedrock : S dep.**	+	+	+	+	+	+	+	+	+	+
**Bedrock : S dep.^{2}**	+	+	+	+	+	**Bedrock : S dep.^{2}**	+	+	+	+	+	+	+	+	+	+
**Bedrock : Temp.**	+	+	+	+	+	**Bedrock : Temp.**	+	+	+	+	+	+	+	+	+	+
**Bedrock : Temp.^{2}**	+	+	+	+	+	**Bedrock : Temp.^{2}**	-	-	-	-	-	-	-	-	-	-
**N dep. : Precip.**	0.00	0.00	0.00	0.00	0.00	**N dep. : Precip.**	0.00	0.00	-	0.00	0.00	0.00	-	-	0.00	0.00
**N dep. : S dep.**	-	**-0.08**	-	-	**-0.04**	**N dep. : S dep.**	-0.09	-0.09	-0.10	-0.09	-0.09	-0.09	-0.10	-0.10	-0.09	-0.09
**N dep. : Temp.**	-0.13	-0.10	-0.12	-0.13	-0.12	**N dep. : Temp.**	-0.06	-0.05	-0.09	-0.07	-0.06	-0.06	-0.07	-0.08	-0.05	-0.08
**Precip. : Temp.**	0.00	0.00	0.00	0.00	0.00	**Precip. : Temp.**	0.00	0.00	0.00	0.00	0.00	0.00	0.00	0.00	0.00	0.00
**S dep. : Precip.**	0.00	0.00	0.00	0.00	0.00	**S dep. : Precip.**	-	-	**0.00**	-	**0.00**	-	**0.00**	**0.00**	**0.00**	**0.00**
**S dep. : Temp.**	-0.15	-0.17	-0.15	-0.14	-0.16	**S dep. : Temp.**	-	-	**0.02**	**0.01**	-	**0.02**	-	**0.02**	-	**0.02**
**df**	35.00	31.00	30.00	33.00	36.00	**df**	27.00	28.00	28.00	28.00	28.00	29.00	27.00	29.00	29.00	29.00
**logLik**	-12759.04	-12763.54	-12764.69	-12761.67	-12758.82	**logLik**	-46330.55	-46329.76	-46330.00	-46330.08	-46330.16	-46329.21	-46331.29	-46329.43	-46329.46	-46329.52
**AICc**	25588.80	25589.66	25589.92	25589.98	25590.41	**AICc**	92715.23	92715.66	92716.14	92716.29	92716.46	92716.56	92716.72	92717.01	92717.08	92717.19
**Adj. R^2**	0.42					**Adj. R^2**	0.13									
**null model AICc**	27487.55					**null model AICc**	93414.60									
**ΔAICc to null m.**	1898.75					**ΔAICc to null m.**	699.37									
**ΔAICc to selected**		0.86	1.12	1.18	1.61	**ΔAICc to selected**		0.42	0.91	1.06	1.23	1.33	1.48	1.78	1.84	1.96

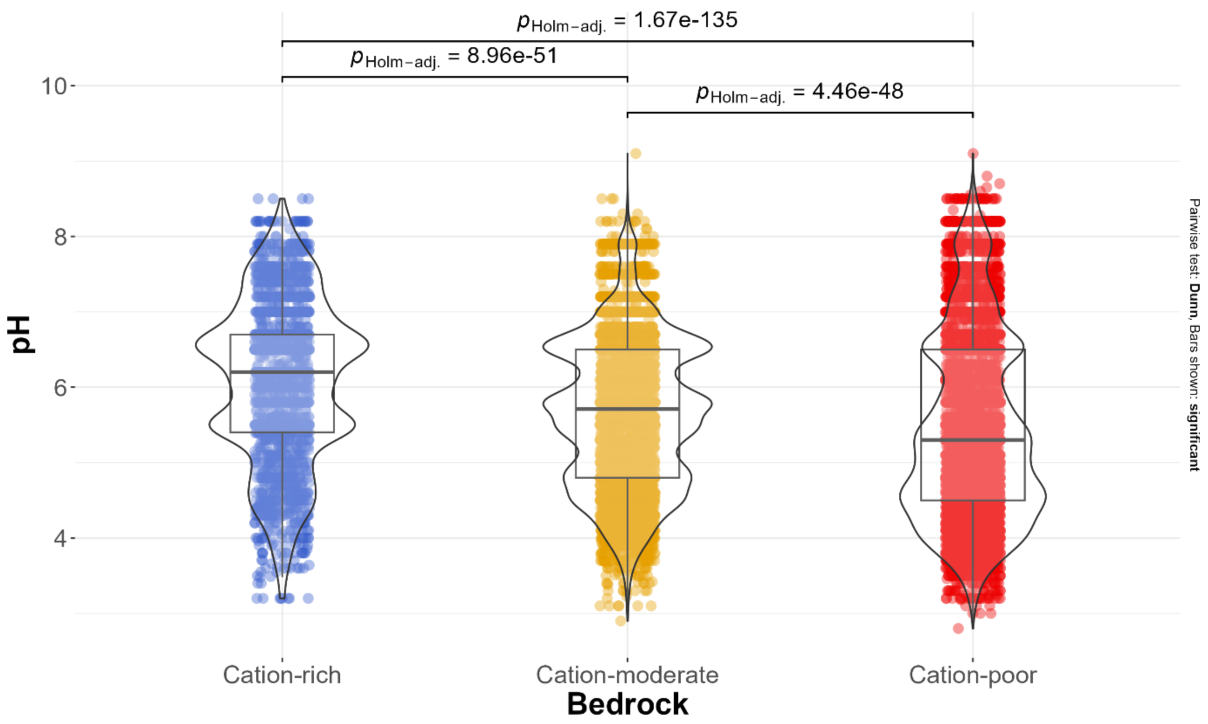

Fig. S6. The box violin plot shows the pH variability within bedrock levels for the US data. The violin plot shows the distribution and density of the data. The embedded boxplot indicates the median and interquartile range (25th–75th percentile). Whiskers extend to values within 1.5 x IQR.

Table S6. Range of Nitrogen Critical Loads as derived for the open and closed canopy vegetation in the study of Simkin et al. 2016 and the range of Nitrogen Critical Loads defined by Bobbink et al. 2022 for vegetation types found in Czechia.

**N critical load range**	**US**	**CZ**
	**Simkin et al. 2016**	**Bobbink et al. 2022**
	kg N ha^{-1} yr^{-1}	
**Open canopy vegetation**	7-10	5-25
**Closed canopy vegetation**	8-20	3-20

References:

Bobbink, R., C. Loran, and H. Tomassen. 2022. Review and revision of empirical critical loads of nitrogen for Europe. Page (R. Bobbink, C. Loran, and H. Tomassen, Eds.). German Environmental Agency, Dessau-Roßlau.

Chuman, T., P. Gürtlerová, J. Hruška, and M. Adamová. 2014. Geochemical reactivity of rocks of the Czech Republic. Journal of Maps 10:341–349. https://doi.org/10.1080/17445647.2013.867418.

Hartmann, J., and N. Moosdorf. 2012. The new global lithological map database GLiM: A representation of rock properties at the Earth surface. Geochemistry, Geophysics, Geosystems 13:1–37. https://doi.org/10.1029/2012GC004370.

Simkin, S. M., E. B. Allen, W. D. Bowman, C. M. Clark, J. Belnap, M. L. Brooksf, B. S. Cade, S. L. Collins, L. H. Geiser, F. S. Gilliam, S. E. Jovan, L. H. Pardo, B. K. Schulz, C. J. Stevens, K. N. Suding, H. L. Throop, and D. M. Waller. 2016. Conditional vulnerability of plant diversity to Atmospheric nitrogen deposition across the United States. Proceedings of the National Academy of Sciences of the United States of America 113:4086–4091. https://doi.org/10.1073/pnas.1515241113.
